# 

**Published:** 1859-02

**Authors:** 


### *Deaths of Medical Men.*

*Death of M. Bérard.*—We learn with regret that M. Bérard, Professor of Physiology at the Faculty of Medicine at Paris, has just died, after an illness which had prevented him from lecturing for the past three years.—*London Lancet.*

At Cambridge, Jan. 4th, Dr. Samuel Sawyer, aged 54.

In New Orleans, Oct. 28th, after a long and painful illness, Dr. J. M. W. Picton, for the past thirty-two years an eminent practitioner in that city, and late Professor of Diseases of Women and Children in the New Orleans School of Medicine.—*Boston Medical Journal.*

We learn as we are going to press that the distinguished physician and pathologist, Dr. Richard Bright, of London, is deceased. He died about the middle of December, after a very short illness. The *Medical Times and Gazette* of December 18th, from which we take this announcement, does not mention the cause of death.—*American Medical Monthly.*

Dr. Canton, of Warsaw, Ala.

Dr. J. S. Duval, of Houston, Texas.

Dr. David Uhl, is reported to have died recently at Bolivia, Venezuela. Dr. Uhl was the same whose name was so unfortunately connected with the Burdell affair at New York. Let the memory of his misdeeds die with him.

*To Subscribers.*—The publisher is endeavoring to straighten the business of the Journal as much as possible, and with that intention will send bills with the present number to all who are in arrears for this volume. Subscribers are asked to remit the amount of their indebtedness before the next issue, when they will be able to take advantage of the advance rates, $2 50, and receipts will be sent then with the April number. After that time, however, we will be compelled to require the full price, $3. As our subscription list is much increased within the last two months, and we are desirous of supplying the perfect volume to all that desire it, we renew our offer to credit 50 cents to any subscriber who will send us the August number, of which we are still short. *Two dollars and the August No.* will be received in payment for the current year.

---

*Hints to Craniographers.*—We have received an excellent article on the subject of Craniography, presented to the Academy of Natural Sciences, of Philadelphia, by Prof. J. Aitken Meigs. The author recommends the publication of catalogues of the various craniological collections, which will enable those interested in that subject to compare the specimens, and make advantageous exchanges of duplicates. Such a movement would, undoubtedly, be productive of much good.

